# Diurnal Variation Induces Neurobehavioral and Neuropathological Differences in a Rat Model of Traumatic Brain Injury

**DOI:** 10.3389/fnins.2020.564992

**Published:** 2020-09-29

**Authors:** Ricardo Jesus Martinez-Tapia, Francisco Estrada-Rojo, Teresita Guadalupe Lopez-Aceves, Veronica Rodríguez-Mata, Armando Perez-Torres, Antonio Barajas-Martinez, Stephany Garcia-Velasco, Perla Ugalde-Muñiz, Luz Navarro

**Affiliations:** ^1^Departamento de Fisiologia, Facultad de Medicina, Universidad Nacional Autonoma de Mexico, Mexico City, Mexico; ^2^Programa Regional de Posgrado en Biotecnologia, Facultad de Ciencias Quimico Biologicas, Universidad Autonoma de Sinaloa, Culiacán, Mexico; ^3^Departamento de Biologia Celular y Tisular, Facultad de Medicina, Universidad Nacional Autonoma de México, Mexico City, Mexico

**Keywords:** traumatic brain injury, diurnal variation, neuronal damage, behavioral tests, circadian rhythms

## Abstract

Traumatic brain injury (TBI) induces two types of brain damage: primary and secondary. Damage initiates a series of pathophysiological processes, such as metabolic crisis, excitotoxicity with oxidative stress-induced damage, and neuroinflammation. The long-term perpetuation of these processes has deleterious consequences for neuronal function. However, it remains to be elucidated further whether physiological variation in the brain microenvironment, depending on diurnal variations, influences the damage, and consequently, exerts a neuroprotective effect. Here, we established an experimental rat model of TBI and evaluated the effects of TBI induced at two different time points of the light–dark cycle. Behavioral responses were assessed using a 21-point neurobehavioral scale and the cylinder test. Morphological damage was assessed in different regions of the central nervous system. We found that rats that experienced a TBI during the dark hours had better behavioral performance than those injured during the light hours. Differences in behavioral performance correlated with less morphological damage in the perilesional zone. Moreover, certain brain areas (CA1 and dentate gyrus subregions of the hippocampus) were less prone to damage in rats that experienced a TBI during the dark hours. Our results suggest that diurnal variation is a crucial determinant of TBI outcome, and the hour of the day at which an injury occurs should be considered for future research.

## Introduction

Traumatic brain injury (TBI) is defined as the alteration of brain function, or other evidence of brain pathology, caused by an external force (Menon et al., 2010). TBI is a severe public health problem: in 2016, the number of new cases of TBI amounted to 27.08 million worldwide (GBD 2016 Traumatic Brain Injury and Spinal Cord Injury Collaborators, 2019). In the United States alone, the Center for Disease Control and Prevention (CDC) reports 1.7 million cases annually, including approximately 275,000 hospitalizations and 52,000 deaths (Faul et al., 2010).

TBI causes two types of brain damage: primary damage is the result of tearing, shearing and rupture of nervous tissue and is considered irreversible; secondary damage is a cascade of pathophysiological processes that could exacerbate the damage of the primary lesion (Maas et al., 2008). Some of these pathophysiological processes, namely excitotoxicity through N-methyl-D-aspartate (NMDA) receptor expression (Estrada-Rojo et al., 2018), oxidative stress (O’Neill and Feeney, 2014), and neuroinflammation (Griffin et al., 2019) are related to diurnal variation in the levels of hormones, neurotransmitters, and metabolic intermediates of cognitive pathways. Fonken et al. (2015) found that microglia isolated from the rat hippocampus during the light phase exhibit significantly greater immune response than those isolated during the dark phase. They also found that rats injected with lipopolysaccharide during the light phase have higher sickness behavior scores and hippocampal cytokine production than rats injected with lipopolysaccharide during the dark phase. Differences in immune response activation could be reflected in social behavioral tests.

Several studies have shown that the time at which a pathological event occurs can influence the extent of the damage. The time of onset of cerebral vascular accidents and transient ischemic attacks occurs most often in the early hours of the morning (Raj et al., 2015; Ripamonti et al., 2017). Animal studies have also shown diurnal variation in ischemia-induced damage. For example, Vinall et al. (2000) reported more significant damage if ischemia was induced in the last hours of darkness, while Beker et al. (2018) reported less damage if ischemia was induced during the mid-dark period.

We have previously reported that recovery from TBI varies depending on the time of the day it occurs. A neurobehavioral test showed that rats with brain trauma induced during the dark hours score better than those traumatized during daylight hours (Martínez-Vargas et al., 2006, 2013; Estrada-Rojo et al., 2018). We hypothesized that general health parameters (e.g., body weight and food intake) and neurobehavioral test performance might also present diurnal variation and correlate with morphological damage in different areas of the central nervous system (CNS). Therefore, in this study, we evaluated the effects of TBI induced at two different time points of the light–dark cycle using a 21-point neurobehavioral scale and the cylinder test, a spontaneous motor test. We also correlated diurnal variation in the responses to TBI with structural damage in specific areas of the CNS.

## Materials and Methods

### Animals

Male Wistar rats (250–300 g; 10–12 weeks) were housed individually in a room temperature of 21 ± 2°C with food and water *ad libitum*. Rats were given at least 15 days to acclimate to the room’s conditions before starting experimentation. All rats were maintained on a 12:12 h light: dark cycle, with lights on either at 08:00 or 20:00 h. Measurements of general health parameters (food intake and body weight) were performed daily at the same time (12:00 h) before and after the induction of TBI. All experiments were conducted according to the recommendations of the Ethics Committee of the Faculty of Medicine, Universidad Nacional Autonoma de Mexico (UNAM) (project, 018/2016; approved April 05, 2016), the Animal Care and Use Committee, and the Mexican Official Regulations (NOM 062-ZOO-1999).

### Experimental Design

Rats were divided into two groups: day and night. Experiments in the day group were performed during the hours of light, and TBI was induced at 13:00 h (5 h after the lights were on); experiments in the night group were performed during the hours of darkness, and TBI was induced at 01:00 h (5 h after the lights were off). Behavioral testing was performed at 24 and 72 h after TBI. In the night group, both, TBI and behavioral testing were performed in a dark room with a red light at < 10 lx to avoid disturbances in the sleep-wakefulness cycle (Stenvers et al., 2016; Zhang et al., 2017). Rats in both groups, day and night, were further divided into Sham (*n* = 10) and TBI subgroups (*n* = 10 per subgroup).

### TBI

Rats were anesthetized inside of an acrylic gas-anesthesia chamber (25 cm × 11 cm × 10 cm). Anesthesia was induced with 4.0% isoflurane (Sofloran^®^Vet, PiSA Agropecuaria, Hgo., Mexico) supplemented with O_2_. Upon loss of righting reflex, each rat was removed from the chamber and placed in a nose cone connected to the same isoflurane supply, now adapted to a stereotaxic device. The concentration of isoflurane was reduced to 3.0% to maintain an appropriate anesthetic depth during surgery. Then, the heads were disinfected with chlorhexidine, and a midline incision was made to expose the skull. Subsequently, the site of trauma (primary motor cortex) was located with the stereotaxic device at coordinates *P* = −2 and *L* = 1.4 (Paxinos and Watson, 1998). Trauma was induced with a pneumatic piston calibrated at 40 psi of pressure and at a depth of 6 mm (Verdugo-Díaz et al., 2017; Estrada-Rojo et al., 2018). After the induction of TBI, isoflurane administration was stopped, and the rat was placed back in its cage. The rat was positioned in a dorsal recumbency to test reflexes until regaining the righting reflex. Subjects in the sham group were only anesthetized.

### Neurobehavioral Scale

Motor deficit after TBI was measured using a previously described 21-point neurobehavioral scale (Hunter et al., 2000). Measurements were taken at baseline (24 h before TBI) as well as at 24 and 72 h after TBI. A blinded observer recorded the neurobehavioral scale score for each rat. The following items were evaluated: paw placement [4 points (pts)], righting reflex (1 pt), horizontal-bar (3 pts), inclined platform (3 pts), rotation (2 pts), visual forepaw reaching (2 pts), contralateral reflex (1 pt), circling (1 pt), motility (2 pts), and general condition (2 pts). The maximum score in healthy rats was set to 21 pts. Although this scale was primarily designed to evaluate cerebral ischemia, cerebral ischemia and trauma share similar pathophysiological mechanisms (Leker and Shohami, 2002). In fact, we have previously used this scale to assess neurological damage after TBI (Martinez-Vargas et al., 2012, 2014; Estrada-Rojo et al., 2018).

### Cylinder Test

Functional recovery is usually assessed with behavioral tests that measure functional deficits; in this study, we measured the motor skills associated with damage to specific brain regions. Several motor control tests indicate trauma- or ischemia-induced brain damage in the cortex sensorimotor area. The cylinder test is widely used in rats to evaluate functional deficits in forelimb activity (Roome and Vanderluit, 2015). To measure spontaneous motor activity and limb use asymmetry after TBI, we used the cylinder test as described previously (Rodgers et al., 2014). Measurements were taken at baseline (24 h before TBI) as well as at 24 and 72 h after TBI. A 15-cm diameter acrylic cylinder was used; the rats were placed inside and their activity was recorded. A blinded observer analyzed the videos at a later time. The number of times the rat placed the left or right leg, or both, on the cylinder wall was scored.

### Tissue Processing and Staining

Rats (*n* = 4, per time point) were deeply anesthetized with sodium pentobarbital (Pisabental^®^, 50 mg/kg i.p., PiSA Agropecuaria, Hgo., México) at 24 or 72 h after induction of TBI. Then, they were perfused transcardially with sodium phosphate buffer, followed by 4% buffered paraformaldehyde. The brains were removed immediately after perfusion and then submerged in the same fixative for at least 24 h. Then, the brains were rinsed with tap water, dehydrated by immersion in ascending ethanol grades, cleared in xylene, and embedded in paraffin with proper orientation to obtain three series of 4-μm-thick sagittal sections, from the midline to the left side of the brain (the TBI zone). The sections were stained with hematoxylin and eosin (HE) to examine the histopathological changes.

### Image Acquisition

Morphological changes were analyzed in four different areas: three subregions of the hippocampus [CA1, CA2/3, and dentate gyrus (DG)] and a perilesional cortex (Cx) area adjacent to the trauma site. We obtained a total of 50 fields with a 40× objective with an area per field calculated previously at 19.700 μm^2^. Images were acquired using a CX31 Olympus microscope equipped with a digital camera and analyzed with Infinity Analyze^®^ software (version 6.3.0).

### Morphometric Analysis

Cell counts were obtained from the Cx in all fields of the three tissue sections. Image analysis was performed to determine the presence of normal neurons (NN), neurons with changes (NCh), and degenerating neurons (DN) according to the criteria established in Table 1. Also, changes in reactivity astrocyte were assessed based on cellular and nuclear size and the chromatin aspect (Garman, 2011); as well, vasculature was straightforwardly observed based on the lumen size compared with its sham subgroup. Average counts were calculated for each rat from three experienced blinded observers. In the three subregions of the hippocampus, the percentage of DN regarding the total number of neurons per field was set on to observe the tissue response to secondary damage. Finally, the counts in the three tissue sections were averaged. FIJI software (v.2.0.0)^1^ was used for cell counting and to determine the field area.

**TABLE 1:** Criteria for cell count based on neuronal morphology.

NN	NCh	DN*
• Large nucleus with open chromatin and a prominent nucleolus • Cell bodies with abundant cytoplasm, variable Nissl substance (rough endoplasmic reticulum) • Shape and size of the cell body	• Collapsed, dark nucleus, and poorly defined nucleolus • Decreased neuronal size (shrunken or contracted) • Basophilic inclusion bodies in the perikaryon • Cytoplasm appears condensed and stains darkly (dark neurons)	• Cell body shrinkage • Loss of Nissl substance • Small/shrunken, darkly stained (pyknotic) nucleus that might eventually undergo fragmentation (karyorrhexis) • Cell membrane rupture Intense eosinophilic staining of the cytoplasm

NN, normal neurons; NCh, neurons with changes; DN, degenerating neurons. *The name and morphological characteristics are based on the work published by Garman, 2011.

### Statistical Analyses

The results are reported as the mean values ± standard error of mean (SEM). Statistical analyses were performed using GraphPad Prism software (GraphPad Software Inc., San Diego, CA, United States). Unless otherwise indicated, data met the assumptions of equal variances (Spearman’s test for heteroscedasticity and homoscedasticity plot). Statistical significance was assessed with two-way analysis of variance and Tukey’s multiple comparisons corrections for food intake, body weight, cylinder test, and neuronal counting, and Kruskal–Wallis and Mann–Whitney *U* tests for neurological scores. Differences were considered to be significant at *p* < 0.05.

## Results

### General Health Parameters Vary Depending Upon the Time of TBI Induction

**FIGURE 1:**
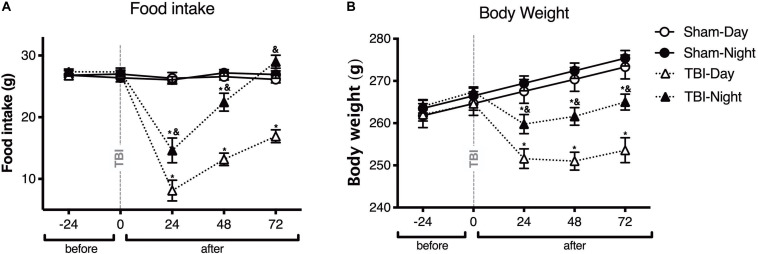
The time of day at which trauma is induced determines the changes in general health parameters in rats. Effect of inducing traumatic brain injury (TBI) at two different time points, i.e., day (white symbols) and night (black symbols), on food intake **(A)** and body weight **(B)** (*n* = 5 per subgroup). Note the significant decrease in food intake and body weight in rats with TBI induced during the day (13:00), an effect that was maintained for up to 72 h after TBI. In rats with TBI induced at night (01:00), the food intake did not differ significantly between the TBI and sham subgroups 72 h after TBI. Data are expressed as the mean ± SEM. Two-way analysis of variance (ANOVA) and Tukey’s test were used as *post-hoc* tests **(A,B)**. *p < 0.05 between TBI and sham groups; ^&^*p* < 0.05 between day and night groups.

To test whether the general health parameters changed depending upon the time of TBI induction in rats, food intake and body weight were measured in both sham and TBI subgroups before and at 24, 48, and 72 h after TBI (Figure 1). Food intake differed in both day and night groups, at 24, 48, and 72 h after the TBI, compared to baseline [*F*_(4,64)_ = 59.05, *p* < 0.0001]. Moreover, there were significant differences between day and night groups [*F*_(3,16)_ = 73.73, *p* < 0.0001]; food intake decreased significantly 24 h after TBI in the day group as compared with the night group (*post-hoc p* < 0.0001). This difference was maintained even after 48 h (*p* < 0.0001), and 72 h (*p* < 0.0001). The night group showed an increased food intake compared with the baseline of the sham subgroup 72 h after TBI; however, this difference was not statistically significant (*p* = 0.38; Figure 1A). Meanwhile, there was a significant decrease in body weight in the day and night groups at 24, 48, and 72 h after TBI [*F*_(4,64)_ = 53.07, *p* < 0.0001]. Similarly, differences in body weight were observed between the day and night groups [*F*_(3,16)_ = 44.69, *p* < 0.0001], with more significant weight loss at 24 h after TBI in the day group than in the night group (*p* < 0.0001). Finally, a tendency to regain body weight was observed between 48 and 72 h after TBI; however, the extent of recovery in body weight did not match that of the sham subgroup (Figure 1B).

### Neurobehavioral Scale and Cylinder Test Scores Vary Depending Upon the Time of TBI Induction

**FIGURE 2:**
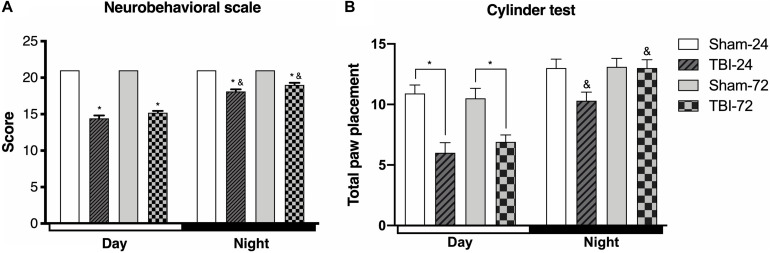
The time of the day at which trauma is induced determines the behavioral response. Differences in the neurobehavioral scale **(A)** and cylinder test **(B)** depending on the time at which traumatic brain injury (TBI) was induced (*n* = 10 per subgroup). Note the significant decrease in both behavioral tests in rats undergoing TBI during the day (13:00), a decrease that continued until 72 h after TBI. The group that underwent TBI at night (01:00) shows a decrease at 24 h after TBI that results in a score that is greater than that in the day group. However, at 72 h, there is a marked increase in the score, which becomes similar to that of the corresponding sham subgroup (cylinder test). Data are expressed as the mean ± SEM. **(A)** Kruskal–Wallis and Mann–Whitney *U* tests. **(B)** Two-way ANOVA and Tukey’s test as *post-hoc* test, *p < 0.05 between TBI and sham groups; ^&^*p* < 0.05 between day and night groups.

Functional changes following TBI were assessed using two neurobehavioral tests (Figure 2). A comparison of the total score 21-point neurobehavioral scale (Figure 2A) showed a decrease in the day group as compared with the sham group at both 24 h (*p* < 0.0001) and at 72 h after TBI (*p* < 0.001). Moreover, the rats with TBI in the night group presented a decrease in the score at 24 h (*p* < 0.0001), and 72 h (*p* < 0.0001), compared with its corresponding sham group. However, the analysis between the day and night group showed significant differences in the total 21-neurobehavioral scale score; the score was better in the night group at both 24 h (*p* < 0.0001) and 72 h (*p* < 0.0001 after TBI than in the day groups.

A detailed description of each specific parameter of the 21-point neurobehavioral scale is shown in Table 2. We found a significant decrease compared to its baseline in the day group at 24 h after TBI in paw placement (*p* < 0.05), horizontal bar (*p* < 0.05), inclined platform (*p* < 0.05), rotation (*p* < 0.05), visual forepaw reaching (*p* < 0.05), circling (*p* < 0.05), and general conditions (*p* < 0.05). At 72 h after TBI, the overall score (as well as the score of five parameters) tended to increase but the differences were not statistically significant. In the night group, at 24 h after trauma there was a significant decrease in only three parameters: horizontal bar (*p* < 0.05), rotation (*p* < 0.05), and general conditions (*p* < 0.05). At 72 h after TBI, only rotation was significantly increased compared to 24 h time point (*p* < 0.05). Other parameters, such as circling, motility, and general condition, tended to decrease compared to the 24-h time point but the differences were not statistically significant. When comparing the day and the night groups at both 24 and 72 h after TBI, we found that paw placement (*p* < 0.05) and rotation (*p* < 0.05) scored better in the night group. Inclined platform (*p* < 0.05) and circling (*p* < 0.05) only scored better and were statistically significant at 24 h after TBI; and horizontal bar (*p* < 0.05) and visual forepaw reaching (*p* < 0.05) were significantly different only at 72 h after trauma. Finally, righting reflex and contralateral reflex were not affected, neither by TBI nor the time it occurred.

**TABLE 2:** Specific parameters of the neurobehavioral scale.

Parameter. Description (Maximum score)	Day (*n* = 10)			Night (*n* = 10)			
		
	Baseline	24 h	72 h	Baseline	24 h	72 h	*p*
**Paw placement.** The animal held lengthways at the edge of bench and each paw placed in turn on the edge of the bench. Each successful paw placement back on bench scored 1 point (4)	4	2.6 ± 0.34*	2.8 ± 0.20*	4	3.7 ± 0.15^&^	3.9 ± 0.10^&^	<0.0001
**Righting reflex**. The animal is placed on its back on palm of the hand. Scored 1 if righted itself (1)	1	1 ± 0.00	1 ± 0.00	1	1 ± 0.0	1 ± 0.0	ns
**Horizontal bar.** Forepaws placed on ribbed bar. Score: 3 if both hindlimbs raised onto the bar, 2 if one hindlimb raised, 1 if animal just hangs and 0 if animal falls off (3)	3	1.9 ± 0.18*	2.1 ± 0.28*	3	2.3 ± 0.21*	2.8 ± 0.13^&^	<0.0001
**Inclined platform.** The animal is placed facing down at an inclination of 45°. Score: 3 if the animal rotates to face “uphill” within 15 s, 2 if it takes 15–30 s, 1 if it takes longer than 30 s and 0 if the animal falls off or remains pointing downwards (3)	3	2.2 ± 0.29*	2.5 ± 0.17*	3	2.9 ± 0.10^&^	2.9 ± 0.10	0.0011
**Rotation**. Animal is held by the base of the tail and rotated clockwise then anticlockwise. Animal should swivel up contralaterally to the direction of rotation. Score 1 for each side (2)	2	0.9 ± 0.10*	1.2 ± 0.20*	2	1.4 ± 0.16*^,&^	2.0 ± 0.00^&,[*dollar*]^	<0.0001
**Visual forepaw reaching.** Ability of animal to reach to bench when held slightly away from it. Score 1 for each successful forepaw placement (2)	2	1.6 ± 0.16*	1.6 ± 0.16*	2	1.7 ± 0.15	2.0 ± 0.00^&^	0.0178
**Contralateral reflex**. Gently touch the animal from the side. Score 0 for a reflex and 1 for no reflex (1)	1	1 ± 0.00	1 ± 0.00	1	1 ± 0.00	1 ± 0.00	ns
**Circling**. Score 1 for non-circling, 0 for circling (1)	1	0.3 ± 0.15*	0.4 ± 0.16*	1	0.9 ± 0.10^&^	0.8 ± 0.13	0.0002
**Motility**. Score 2 for normal motility, 1 rocking and unsteady and 0 if immobile (2)	2	1.9 ± 0.10	1.6 ± 0.16*	2	1.9 ± 0.10	1.5 ± 0.17*	0.0104
**General condition.** Score 2 if normal (good coat condition, alert, moving about), 1 unkempt (e.g., dirty coat, hunched posture, aggressive) and 0 if thin, weak and poor muscle tone (2)	2	1 ± 0.00*	1.0 ± 0.00*	2	1.3 ± 0.15*	1.1 ± 0.10*	<0.0001
Total (21)	21	14.4 ± 0.40*	15.2 ± 0.25*	21	18.1 ± 0.27*^,&^	19 ± 0.29*^,&^	<0.0001

*Data are expressed as mean ± SEM. Kruskal-Wallis and Mann-Whitney U-test were used as post-hoc test. ^∗^*p* < 0.05 versus sham; ^&^*p* < 0.05 between day and night; ^[*dollar*]^*p* < 0.05 versus 24 h.*

To test spontaneous exploration and limb use asymmetry after TBI, we used the cylinder test (Figure 2B). In the day group, there were differences in the time after the induction of the trauma [*F*_(3,54)_ = 11.84, *p* < 0.0001], at both 24 h (*p* < 0.0001) and 72 h (*p* < 0.0001) after TBI, when compared with the sham subgroup. However, the rats with TBI in the night group did not show statistically significant differences in total paw placement at 24 h (*p* = 0.057) or 72 h (*p* = 0.999) compared with the corresponding sham subgroup. Finally, there were significant differences in spontaneous exploration between both the day and night groups [*F*_(1,18)_ = 53.93, *p* < 0.0001]; compared with the day group, the score was higher in the night group at both 24 h (*p* = 0.0004) and 72 h (*p* < 0.001) after TBI.

A detailed analysis of limb use asymmetry was performed (Supplementary Figure S1). Contralateral limb use (right paw) was significantly different between the TBI and sham subgroups [*F*_(3,54)_ = 7.208, *p* = 0.0004, *post-hoc: p* = 0.0211] of the day group, at 72 h after TBI. In the night group, a difference in limb use asymmetry was found only at 24 h after TBI (*p* = 0.0271), but not at 72 h (*p* = 0.418), compared with the corresponding sham subgroup. There was also a significant difference in limb use asymmetry between the day and night groups [*F*_(1,18)_ = 24.19, *p* = 0.0001; Supplementary Figure S1A], however, *post-hoc* analysis showed no significant difference in the TBI subgroups in both day and night groups at 24 h (*p* = 155) and 72 h (*p* = 0.0750).

Finally, time of TBI induction had no significant effect on the use of the ipsilateral limb (left paw) [*F*_(3,54)_ = 1.146, *p* = 0.338]. Nevertheless, in the day group, *post-hoc* analysis showed a significant difference between the TBI and sham subgroups at 24 h (*p* = 0.0311). However, no such significant difference was found in the night group. Differences between the day and night groups were found [*F*_(1,18)_ = 8.201, *p* = 0.0103; Supplementary Figure S1B] only at 72 h after TBI (*p* = 0.0012).

### The Time of Day at Which TBI Occurs Determines the Degree of Cell Injury in the Perilesional Zone (Motor Cortex) of the CNS

**FIGURE 3:**
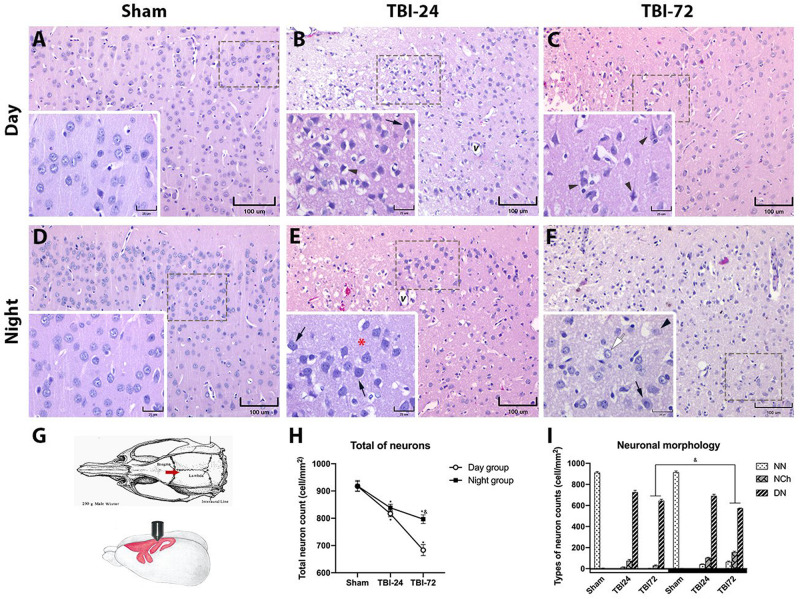
Histopathology of the perilesional motor cortex reveals less morphological damage and fewer degenerating neurons (DN) in the group with traumatic brain injury (TBI) induced at night. Photomicrographs of the cerebral motor cortex in the day **(A–C)** and night groups **(D–F)** (*n* = 4 per column). Schematic representation of the rat motor cortex where TBI was induced (**G**; image modified from Paxinos and Watson, 1998). An increase in neuronal basophilia (arrow) was observed 24 h after TBI in the lamina 3 (external pyramidal cell layer) of the motor cortex, along with vasodilation (*v*) in both day **(B)** and night **(E)** groups. Note the presence of DN (arrowhead) in the day group and the better preservation of the neuropil (red asterisk) in the night group. At 72 h after TBI, neuronal changes were maintained with the persistence of DN (arrowhead) in the day group **(C)**. In the night group **(F)**, the neurons appeared normal (white arrow) and with changes (arrow); the perineuronal spaces and vasodilation were more prominent in the group with TBI induced during the day **(C)** than in the group with TBI induced at night **(F)**. The total neuron count in the perilesional zone to trauma **(H)**, note the similar decrease in both groups at 24 h after TBI, and the large neuronal loss at 72 h after trauma in the day group. Cell count per mm^2^, according to the neuronal morphology classification in Table 1 **(I)**. Data are expressed as the mean ± SEM. Two-way ANOVA and Tukey’s test as *pos-hoc* test. *p < 0.05 between TBI and sham groups; ^&^*p* < 0.05 between day and night groups. Bars (**A–F**, 100 μm; bars in the insets, 25 μm).

To determine whether the damage evaluated by neurobehavioral tests correlated with morphological damage, formalin-fixed paraffin-embedded brain tissue sections were obtained and stained with HE. The perilesional zone of the site of TBI (motor cortex) was examined with a light microscope. The histopathological observations are presented in Figure 3. The figure legends describe the histopathological changes at 24 and 72 h after TBI in the day group (Figures 3A–C) and night group (Figures 3D–F). Findings such as vascular congestion, vasodilation, hemorrhage and necrosis in the perilesional zone at TBI are not shown so as to highlight the neuronal changes. The total neuron count (Figure 3H) in the perilesional zone of the site of TBI (motor cortex) varied significantly with time [*F*_(2,12)_ = 173.9, *p* < 0.0001] and experimental groups (day group vs. night group) [*F*_(1,12)_ = 33.97, *p* < 0.0001]. The total neuron count in the TBI subgroup at 24 h in both the day group (*p* < 0.0001) and the night group (*p* = 0.0008) was significantly lower than that in the corresponding sham subgroups. However, at 72 h, the total decrease in neurons was maintained in the night group (*p* = 0.0793) but decreased further in the day group (*p* < 0.0001). Neuron count between the day and night groups showed no significant differences at 24 h after TBI (*p* = 0.6144), but statistically significant differences were observed at 72 h after TBI (*p* < 0.0001). NN count was considerably low, while NCh and DN counts were high at 24 and 72 h after TBI (Figure 3I) in both day and night groups.

NN count varied significantly with time (24 and 72 h) [*F*_(2,12)_ = 10,748, *p* < 0.0001] and experimental groups (day and night groups) [*F*_(2,12)_ = 29.15, *p* = 0.0002]. At 24 h after TBI, a significant decrease in NN was observed in both in the day group (*p* < 0.0001) and the night group (*p* < 0.0001) but no significant differences were observed between the two groups. However, at 72 h after TBI, there was a significant difference between the day and night groups (*p* = 0.0005).

With regard to NCh count, a significant effect was observed in time [*F*_(2,12)_ = 105.2, *p* < 0.0001] and experimental groups [*F*_(1,12)_ = 80.46, *p* < 0.0001]. There were significant differences in NCh counts between TBI subgroups of day and night groups at 72 h after TBI (*p* < 0.0001), but not at 24 h (*p* = 0.1069).

Finally, DN count, also varied significantly with time [*F*_(2,12)_ = 105.2, *p* < 0.0001] and experimental groups [*F*_(1,12)_ = 80.46, *p* < 0.0001]. There were significant differences in DN counts between TBI subgroups of day and night groups at 72 h after TBI (*p* = 0.0058), but not at 24 h (*p* = 0.2895).

### TBI-Induced Neuronal Damage Is Specific to Certain Hippocampal Subregions and Follows a Diurnal Pattern

**FIGURE 4:**
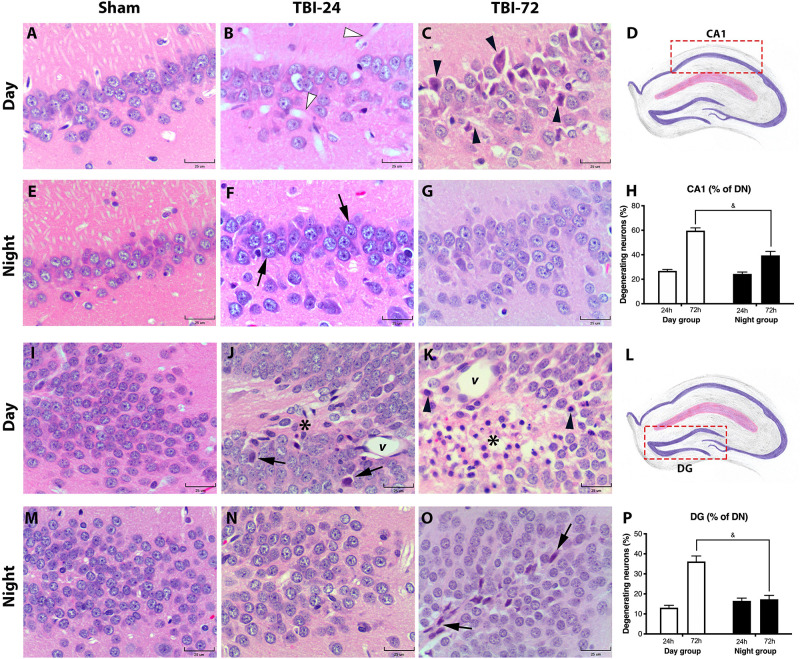
Histopathology of the hippocampal subregion CA1 and DG reveals less morphological damage and a lower percentage of degenerating neurons (DN) in the group with traumatic brain. Injury (TBI) induced at night. CA1 hippocampal subregion in the day group **(A–C)** and the night group **(E–G)**. At 24 h after TBI, the day group **(B)** did not show remarkable changes in the neuronal morphology, except large vasodilation (white arrowhead) which was not observed in the night group **(F)**. However, in the night group, the layer of pyramidal neurons had more basophilia (arrow). At 72 h after TBI, the day group **(C)** had more dead pyramidal neurons, characterized by pyknotic nuclei, retraction, and cytoplasmic eosinophilia (arrowhead), compared with the well-preserved pyramidal neurons in the night group **(G)**. In both the day and night groups, a large dispersion of the pyramidal neuron layer was observed both at 24 h **(B,F)** and 72 h **(C,G)** after TBI compared with their respective sham subgroup **(A,E)**. Analysis of the percentage of DN **(H)** showed an increase in both the day and night groups at 24 h after trauma. However, in the day group at 72 h after TBI, the percentage of DN was higher than that in the night group. DG in the day group **(I–K)** and in the night group **(M–O)**. At 24 h after TBI, the day group **(J)** showed few DN (arrow) in the granular layer, vasodilation (*v*), and astrogliosis (asterisk). In contrast, 24 h after TBI, the DG of the night group **(N)** showed few histological changes in the granular layer. At 72 h after TBI **(K)**, morphological changes in the day group were accentuated, particularly astrogliosis (asterisk), and vasodilation (*v*), and DN (arrowhead). The night group **(O)** had few shrunken and basophilic neurons (arrow) in the granular layer. The percentage of DN **(P)** showed a large increase in the day group, particularly at 72 h after TBI; however, in the night group, the total percentage of DN remained the same at 24 and 72 h. The difference at 72 h between the day group and night groups was significant. Diagrams of the CA1 **(D)** and DG **(L)** areas where the photomicrographs from the tissue sections were acquired, and the percentage of DN was determined. **(H,P)** Data are expressed as the mean ± SEM. Two-way ANOVA and Tukey’s test as *post-hoc* test. ^&^*p* < 0.05 between day and night groups. HE staining (bars, 25 μm).

To study the histopathological extent of secondary brain damage after TBI, we performed morphometric analysis of DN in three different hippocampal subregions–*Cornus Ammonis* 1, 2, 3 (CA1, 2, and 3) and DG (Figure 4 and Supplementary Figure S2). The histopathological description is found in their respective figure caption. Time [*F*_(1,8)_ = 117, *p* < 0.0001] and experimental group [*F*_(1,8)_ = 26.14, *p* = 0.0009] had a significant effect on the percentage of DN in CA1 (Figure 4H). There were no significant differences in DN at 24 h after TBI between day and night groups (*p* = 0.8555); however, at 72 h after TBI, the day group had a higher percentage of DN than the night group (*p* = 0.0009). Meanwhile, the percentage of DN in the DG (Figure 4P) differed significantly with time [*F*_(1,8)_ = 37.98, *p* = 0.0003] and experimental group [*F*_(1,8)_ = 15.86, *p* = 0.0040]. There were no significant differences in DN at 24 h after TBI between day and night groups (*p* = 0.6157); however, at 72 h after TBI, the day group had a higher percentage of DN than the night group (*p* = 0.0006). Finally, the percentage of DN in the hippocampal CA2/3 (Supplementary Figure S2) was considerably high between 24 and 72 h after TBI in the day group (*p* = 0.0173); however, this increase was not observed in the night group (*p* = 0.3599). Moreover, there were no significant differences between the two groups at 24 h (*p* = 0.8516) or 72 h (*p* = 0.5243) after TBI.

## Discussion

Circadian variations in an organism’s physiological variables are essential because they determine the response to environmental events, for example, an injury. However, there is limited research on how these variations determine the degree of injury after TBI. In this study, we showed that general health parameters and scores in two different neurobehavioral tests vary depending on the phase of the light–dark cycle at which TBI occurred. Moreover, these changes correlated with histopathological damage in specific areas of the CNS.

After a TBI, a series of events occur locally and in various systems, at both behavioral and cognitive levels. These events impact the general state of the organism because they can cause loss of the appetite, so that the body’s reserves are available, and therefore its use can lead to weight loss (Martínez, 2011). In a previous study, rats with fluid percussion injury showed a significant decrease in the food intake and body weight 10 days after injury; however, the time of trauma was not considered in the analysis (Moinard et al., 2005). Our results indicated a decline in food intake at 24 h in rats with TBI induced at night, and for up to 72 h in rats with TBI induced during the day. Moreover, rats with TBI induced at night had less weight loss, which could be indicative of a neuroprotective effect of daytime variation. This weight loss has been associated with an increase in hyperglycolysis in the first 24 h after trauma, during which, stored fats become the primary source of energy and proteins are preserved until late in the process (Brooks and Martin, 2015). However, other reports on critical patients have described that numerous metabolic processes might combine to induce a hypermetabolic state characterized by a marked negative nitrogen balance; moreover, the rate of lean tissue loss is 2–3 times higher than that during starvation and lipolysis decreases as glucose intolerance develops, consequentially raising insulin levels (Burke et al., 1979; Dabrowski and Rombeau, 2000). Our results suggest that this hypermetabolic state is more severe in animals that developed TBI during the day.

Several studies have demonstrated that motor deficits after a TBI can occur in both focal lesions and diffuse models (Lindner et al., 1998; Crane et al., 2012; Hehar et al., 2015). Our study results show failures in the motor skills of rats with TBI, according to a modified 21-point neurobehavioral scale (Hunter et al., 2000), which evaluated the motor aspects, special perception, and reflexes, and on the cylinder test, which examined motor cortex damage (Roome and Vanderluit, 2015). Interestingly, statistically significant differences in motor deficits were found between the day and night groups at 24 and 72 h after TBI. Nevertheless, our detailed parameter analysis of the 21-point neurobehavioral test showed that some parameters improved over time, regardless of the moment at which we induced the trauma, such as paw placement, horizontal bar, inclined platform, rotation, and visual forepaw reaching. On the other hand, although there were parameters whose scores were decreased, such as cycling, motility and general conditions, these differences were not statistically significant. We consider that these changes are related to a diffuse axonal injury (DAI) generated after TBI. In previous works, it has been reported that neuronal body loss (which we observed in this work) is related to white matter atrophy due to DAI (Armstrong et al., 2016). Diurnal variation in parameters depending of time at which TBI was induced may be related to changes in neurotransmitters such as GABA; for example, some studies have shown the diurnal variation in inhibitory synapses, which increase gradually in the active phase (night) in the mouse somatosensory cortex (Jasinska et al., 2014, 2015). Righting reflex and contralateral reflex were not affected by either TBI or its time of occurrence. The damage caused in our TBI model was in M1, an area that does not emit the upper commands that affect these reflexes. The parameters corresponding to the reflexes are difficult to change since, as we know, the final integration occurs in the spinal cord, and in our model, both the upper commands and those of the spinal cord itself were not affected by TBI (Møller, 2011).

The cylinder test examines the damage to the sensorimotor cortex due to the TBI. In this study, the placement of the paws or the predominance of one when touching the cylinder is a good measure of damage to the sensorimotor cortex of the anterior limb and helps detect motor deficits. Moreover, our results of the analysis of classic forelimb asymmetry (Supplementary Figure S1) showed differences between the day and night groups, when evaluated at 24 and 72 h after TBI, which indicates behavioral deficits most fine and consistent (Farr and Whishaw, 2002; Tennant et al., 2010). The 21-point neurobehavioral test showed a significant difference between the groups undergoing TBI during the daytime hours as compared to those with TBI induced at night; additionally, there was a significant difference between both day and night groups evaluated at 24 and 72 h after TBI.

During TBI, the damage can be transmitted as a frontal-to-posterior gradient via a DAI process, but with a greater tendency to damage the frontal pole (Eierud et al., 2014). This reduces the cortical control of higher-order motor functions (Tsenter et al., 2008; Rosenbaum and Lipton, 2012). Our neurobehavioral test results are consistent with those reported in human patients who have slow, asymmetric walking, shorter steps, and more significant mediolateral sway (Ozolins et al., 2016). Additionally, reports indicate balance and subtle motor alterations after TBI (Neumann et al., 2009; Finnanger et al., 2013). However, balance is a complex function, that involves both motor and sensory aspects; additionally, cerebellum might be involved. Moreover, the interpretation of results should consider the fact that the rats use two pairs of limbs for walking, which might compensate for the imbalance caused by neuronal damage (Namdar et al., 2020). Our motor deficiency results also correlate with the count of DN in both the cortex and the hippocampus.

The night group demonstrated less tissue damage in the perilesional motor cortex area, with a greater count of total neurons and fewer DN at 72 h after TBI, compared to the day group. Concerning the pathophysiological complexity after TBI (Giza and Hovda, 2014), our results are consistent with a previous report on variation in NMDA receptor expression in the cerebral cortex through the day, which correlates with a better outcome after a TBI (Estrada-Rojo et al., 2018). Moreover, the resident microglia at the sensory-motor cortex have less branching of cytoplasmic extensions during daylight hours (Hayashi et al., 2013), but a comparatively higher and longer cellular processes and branching points at night (Takayama et al., 2016). These morphological changes suggest a daytime pre-pro-inflammatory microenvironment in the cortex or variations in microglia-mediated inflammatory-immune responses. In the hippocampus, heterogeneity in the cellular response for the CA1, CA2/3, and DG regions after TBI has been described in controlled cortical impact models and in a closed head injury model (Grady et al., 2003; Anderson et al., 2005; Mao et al., 2013; Tsuda et al., 2016), the latter of which is similar to our TBI model. However, none of these previous models considered the effect of diurnal variations at the time of TBI.

Our histopathological findings demonstrated a highly significant neuronal loss, measured through DN percentage, in the CA1 and DG hippocampal subregions in the day group. We consider that the result in the CA1 could be related to a relatively high oxidative state during the day in the hippocampal CA1 pyramidal neurons (Naseri Kouzehgarani et al., 2020). Interestingly, we found that the percentage of DN in the DG did not increase during the night phase, even at 72 h after trauma, which could be related to the presence of microglia in this area, where they could exert an immunoregulatory function (Savchenko et al., 1997; Appel et al., 2018; Tan et al., 2020). In contrast, our results showed that the hippocampal CA3 subregion presented an equal increase in DN in both day and night groups. Previous reports have established a higher susceptibility of the CA3 region in the CCI model (Anderson et al., 2005) and mathematics models (Mao et al., 2013) suggesting that it could be due to a biomechanical component following trauma. We consider that CA3 presents unique and functional specializations made up of DG mossy fiber inputs and broad axon collateral fibers between CA3 neurons. This creates not only a highly interconnected net but also excitable, which could make it especially susceptible, regardless of the time of day of injury. Nevertheless, this is the first report of variation in susceptibility in the hippocampal subregions, depending on the time at which a TBI is induced. Moreover, depending on the light–dark cycle, pathological changes may be minor when TBI occurs during the dark phase. This effect could be physiologically explained as circadian variations of the expression of pro-inflammatory factors that cause a diurnal difference in the inflammatory response in the CNS (Fonken et al., 2015; Martínez-Tapia et al., 2020).

Numerous studies show that there is diurnal variation in the presentation of acquired brain damage as in the onset of cerebral vascular events (Raj et al., 2015; Ripamonti et al., 2017). In animal models of ischemia, a time-dependent variation in the damage caused by ischemia. Our data are in line with those reported by Beker et al. (2018), who found less severe neuronal damage, infarct volume, brain swelling, and neurological scores with increased neuronal survival in animals subjected to ischemia during darkness compared to the early light period. However, our data are not in line with those of Vinall et al. (2000), who found more damage at the end of the dark period. In the case of TBI, it is clear that the time of occurrence of this event depends on the activities of the subjects. However, recognizing that there is a differential response depending on the time of occurrence and identifying the systems involved in this response, will provide a novel approach to treat TBI (Martinez-Vargas et al., 2019).

## Conclusion

In conclusion, our results indicate that circadian characteristics of the microenvironment in the area at which TBI occurs are important and influence the outcome of sensorimotor functions. It is important to conduct more experiments on the cellular and molecular characteristics of the microenvironment at time of TBI. The variation in the excitatory and inhibitory neurotransmitters and their receptors, the immunological function of the microglia and astrocytes, and oxidative stress could be main factors that determining the degree of damage or tissue preservation after TBI. However, we consider that our results and published literature corroborate our hypothesis.

## Limitations

Our results show that the morphological damage induced by TBI depends on the phase of the light–dark cycle in which it occurs. Furthermore, we found that the damage in the perilesional zone is correlated with behavioral performance, less morphological damage, and better behavioral performance in rats that experienced a TBI during the dark hours compared with those injured during the light hours. Nevertheless, we consider that further research is needed on TBI and diurnal variation, and we are conscious that the findings on this study have to be seen in the light of some limitations. We performed the TBI model in the motor cortex. However, we consider the hippocampus a structure in which we could analyze the extent of secondary damage. We believe that memory tests could offer us a more comprehensive approach to hippocampal behavior after trauma and not just histopathological evaluation. We analyzed the hippocampus because it is a structure that is very susceptible to damage from almost any TBI, and given its functions, it is important to analyze the changes it undergoes. In the future, we consider performing an analysis using memory tests to get a better understanding of our results. However, these tests go beyond this paper’s scope since they are very time-consuming and require a more in-depth analysis.

Indeed, we are aware that our findings do not offer an explanatory mechanism for the differences we found. We have not analyzed the neurotransmitter systems involved, and whether the hormonal and immune responses are participating in this diurnal variation in the damage–neuroprotection balance. Our future works will focus on these mechanisms, mainly on the difference of the neuroinflammatory response of the microglia, as we highlighted in a previous review published by our group (Martínez-Tapia et al., 2020). While it is true that there is still scope for investigation, the limitations of this research point toward topics to be addressed in the future.

## Data Availability Statement

The raw data supporting the conclusions of this article will be made available by the authors, without undue reservation, to any qualified researcher.

## Ethics Statement

The animal study was reviewed and approved by the Ethics Committee of the Faculty of Medicine, Universidad Nacional Autónoma de México (UNAM) (project, 018/2016; approved April 05, 2016).

## Author Contributions

RJM-T performed the anesthesia of animals and the behavioral experiments. FE-R induced the TBI. TGL-A processed and obtained the brains. VR-M and AP-T processed and stained the tissue sections. AP-T made the histopathological sections. AB-M, SG-V, and PU-M did the cell counting in all tissue sections. LN performed the statistical analysis. RJM-T and LN designed the project. All authors contributed to the article and approved the submitted version.

## Conflict of Interest

The authors declare that the research was conducted in the absence of any commercial or financial relationships that could be construed as a potential conflict of interest.

## Footnotes

1 https://imagej.net/Fiji
