# Supplementary material for: Diurnal Variation Induces Neurobehavioral and Neuropathological Differences in a Rat Model of Traumatic Brain Injury
Source: Front Neurosci. 2020 Sep 29;14:564992. doi: 10.3389/fnins.2020.564992 (PMC7550533; doi:10.3389/fnins.2020.564992)
Supplement: Supplementary file 1 [file Data_Sheet_1.PDF]

Supplementary Material

1.1 Supplementary Figures

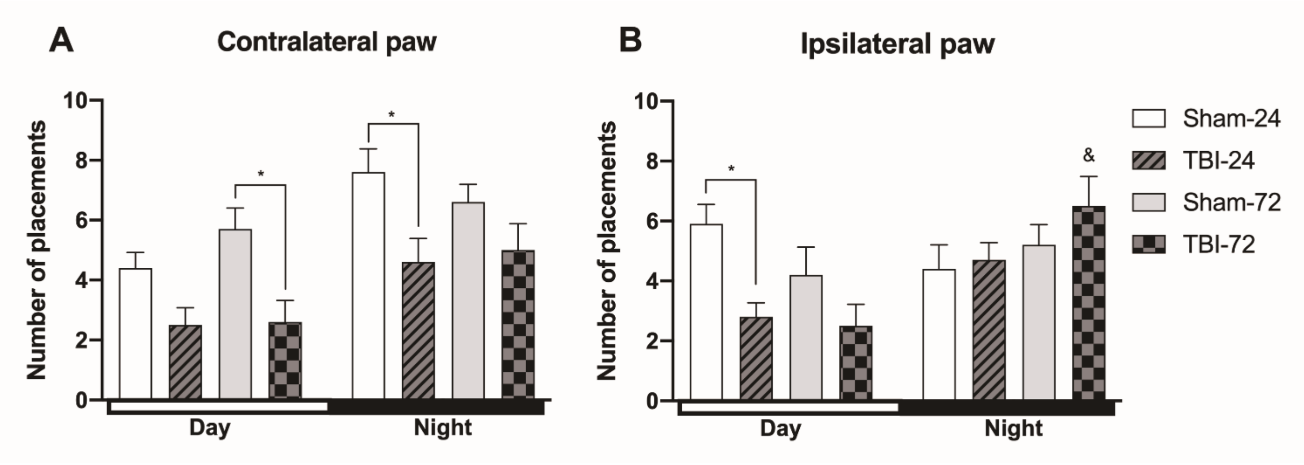

Supplementary Figure 1. Individual paw evaluation of the cylinder test revealed differences in the response after traumatic brain injury (TBI). Contralateral paw refers to the right leg (**A**) and ipsilateral paw refers to the left leg (**B**). The graph of the contralateral paw (right paw) shows a considerable decrease in the number of paw placements at 72 h after TBI in the day group (13:00). In rats with TBI at night (01:00) the number of paw placements was decreased at 24 h after TBI. The graph of the ipsilateral paw (left paw) in the day group showed a decrease in the number of paw placements at 24 after TBI. However, the night group showed a tendency for compensation at 72 h after TBI, observing a significant increase concerning the sham subgroup. Data are expressed as the mean ± SEM. Two-way ANOVA and Tukey's test as *post-hoc test.* *$p < 0.05$ between TBI and sham groups, $^{\&}p < 0.05$ between day and night groups.

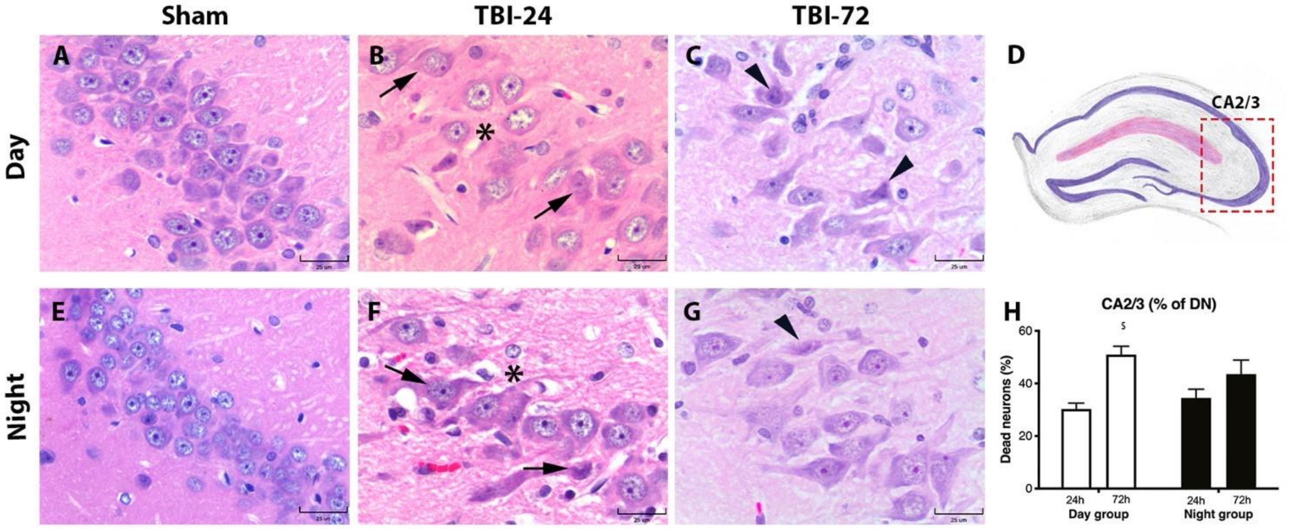

Supplementary Figure 2. Histopathology of the hippocampal subregion CA2/3 reveals no differences in the morphology and the same percentage of degenerating neurons (DN) between the both day and night groups. The hippocampal CA2/3 region of the day (**A-C**) and night groups (**E-G**). Pyramidal neurons showed similar changes at 24 h after TBI in both the day (**B**) and night (**F**) groups such as: increment of neuronal cytoplasmic basophilia (arrow) and soma size, with visible and elongated neurites and loose of neuropil density (asterisk). These changes were maintained even at 72 h after TBI in the day group (**C**) with adding the presence of DN (arrowhead). However, in the night group (**G**), note the well-preserved of neuronal morphology observed. Diagram of the CA2/3 (**D**) area where the photomicrographs were taken and the percentage of DN was determined. (**H**) Data are expressed as the mean ± SEM. Two-way ANOVA and Tukey's test as post-hoc test. $^{\$}$ $p < 0.05$ between 24 and 72 h. HE staining (bars, 25 μm).
